# Selinexor in combination with standard chemotherapy in patients with advanced or metastatic solid tumors

**DOI:** 10.1186/s40164-021-00251-0

**Published:** 2021-12-29

**Authors:** Kyaw Z. Thein, Sarina A. Piha-Paul, Apostolia Tsimberidou, Daniel D. Karp, Filip Janku, Siqing Fu, Vivek Subbiah, David S. Hong, Timothy A. Yap, Jatin Shah, Denái R. Milton, Lacey McQuinn, Jing Gong, Yanyan Tran, Brett W. Carter, Rivka Colen, Funda Meric-Bernstam, Aung Naing

**Affiliations:** 1 Department of Investigational Cancer Therapeutics, The University of Texas MD Anderson Cancer Center, Houston, TX USA; 2 Division of Hematology and Medical Oncology, Oregon Health and Science University/Knight Cancer Institute, 3181 SW Sam Jackson Park Rd, Mail Code: OC14HO, Portland, OR 97239 USA; 3 Karyopharm Therapeutics, Newton, MA USA; 4 Department of Biostatistics, The University of Texas MD Anderson Cancer Center, Houston, TX USA; 5 Department of Thoracic Imaging, Division of Diagnostic Imaging, The University of Texas MD Anderson Cancer Center, Houston, TX USA; 6 Department of Diagnostic Radiology, The University of Texas MD Anderson Cancer Center, Houston, TX USA

**Keywords:** Selinexor (KPT 330), Carboplatin, Irinotecan, FOLFIRI, Doxorubicin and cyclophosphamide, Capecitabine and oxaliplatin (XELOX)

## Abstract

Selinexor, an oral selective inhibitor of nuclear export (SINE), was demonstrated to hinder the DNA damage repair (DDR) system by reducing DDR proteins while enhancing the killing of cancer cells by DDR-based therapeutics in vivo studies. In this single-center, multi-arm phase 1b study, selinexor with carboplatin, doxorubicin and cyclophosphamide (DC), irinotecan with fluorouracil and folinic acid (FOLFIRI), irinotecan, and capecitabine and oxaliplatin (XELOX), were employed as separate parallel arms. Eligible patients have relapsed/ metastatic refractory solid tumors following standard therapy or addition of selinexor to systemic therapy was appropriate. Nineteen patients were treated in the 5 arms. Tumor types included were colorectal (n  = 3), breast (n  = 3), neuroendocrine (n  = 2), ovarian (n  = 2), and pancreas cancers (n  = 2). All patients developed one treatment-related adverse events (TRAE). The most prevalent TRAE were thrombocytopenia (84%), nausea (68%), leukopenia (68%), neutropenia (63%), and fatigue (58%). The common grade 3/4 TRAE were neutropenia (42%), leukopenia (26%), and hyponatremia (21%). Three patients had dose-limiting toxicities (DLT) in 3 separate arms. Fourteen patients were evaluable for response. Although no patients achieved complete or partial response (CR or PR), seven patients attained stable disease (SD). Disease control rate (DCR) was 14%. The combination of oral selinexor with different standard chemotherapies showed limited clinical activity despite toxicity and DLT prevented further dose escalation. Optimizing supportive care, the utility of growth factors, and aggressive measures on antiemetics strategies remain tangible.

*Trial registration* ClinicalTrials.gov Identifier: NCT02419495. Registered 14 April 2015, https://clinicaltrials.gov/ct2/show/NCT02419495). Sponsor(s): Karyopharm Therapeutics

**Supplementary Information:**

The online version contains supplementary material available at 10.1186/s40164-021-00251-0.

To the editor,

Selinexor (KPT-330) is an oral selective inhibitor of nuclear export (SINE) and was shown to hinder the DNA damage repair (DDR) system by diminishing DDR proteins expression while enhancing the killing of cancer cells by DDR-based therapeutics in vivo preclinical models [1–3]. Previous early phase studies demonstrated the modest activity of single agent selinexor in patients with solid tumors [4–6]. In vivo studies of selinexor in combination with different chemotherapeutics demonstrated synergistic activity [3, 7, 8]. An open-label, multi-arm phase 1b study utilizing selinexor in combination with different standard chemotherapies was conducted at MD Anderson Cancer Center to evaluate the safety and tolerability of the combination. Herein, we report results from 5 closed separate arms of the study.

Eligible patients were adult (age  ≥ 18 years) patients with histologically documented, relapsed/metastatic refractory solid tumors following standard therapy or adding selinexor to systemic therapy was appropriate. The primary aim was to evaluate the safety and tolerability of selinexor when given in combination with different standard chemotherapies and the secondary aim was to determine the preliminary antitumor activity [DCR (CR  +  PR  +  SD  ≥ 6 months), and progression-free survival (PFS)].

Nineteen patients with relapsed/refractory solid tumors were enrolled between July 2015 and January 2017. The demographic and clinical characteristics of all patients enrolled are summarized in Table 1. No patients are currently in the study where the majority of patients withdrew from the study due to progression of disease while 5 patients withdrew due to clinically intolerable treatment-emergent adverse events (TEAE). All patients experienced at least one TEAE and TRAE (Table 2 and Additional file 1: Table S2). The commonest TRAE were thrombocytopenia (84%), nausea (68%), leukopenia (68%), neutropenia (63%), fatigue (58%), vomiting (53%), and anemia (53%). The most prevalent grade 3/4 TRAE were neutropenia (42%), leukopenia (26%), hyponatremia (21%), anemia (16%), and thrombocytopenia (16%). Three patients experienced DLTs; a patient dosed at selinexor 60 mg twice weekly (BIW) with DC reported grade 3 leukopenia and grade 4 neutropenia; a patient receiving selinexor at 40 mg once weekly (QW) with FOLFIRI reported grade 3 febrile neutropenia (FN); and a patient receiving selinexor at 40 mg QW with XELOX reported grade 3 diarrhea. Four patients were reported to have serious adverse events (SAEs). One patient receiving selinexor with DC had an SAE from treatment-related grade 3 FN. Two patients in the combination of selinexor with FOLFIRI had SAEs; one patient had treatment-related grade 3 FN while the other had treatment-unrelated grade 2 pancreatitis. One patient receiving selinexor with XELOX had an SAE from treatment-related grade 3 diarrhea and treatment-unrelated grade 3 dyspnea and skin infection. No patients died during the study.

**Table 1:** Patients baseline demographics and disease characteristics

Characteristic	Selinexor 60 mg QW/BIW + carboplatin 6 AUC Q3W (*N* = 6)	Selinexor 60 mg QW/BIW + doxorubicin 60 mg/m^2^ + cyclophosphamide 600 mg/m^2^ Q3W (*N * = 4)	Selinexor 40 mg QW + FOLFIRI^a^ (*N * = 3)	Selinexor 60 mg QW + irinotecan 125 mg/m^2^ D1 and 8 Q3W (*N* = 3)	Selinexor 40 mg QW + XELOX^b^ (*N* = 3)	All patients (*N* = 19)
Age at consent (years)						
Median range	56.1 (31.2–76.1)	61.7 (23.6–64.9)	49.9 (38.1–70.7)	67.8 (57.9–72.3)	65.9 (51.8–72.8)	60.6 (23.6–76.1)
Gender, *N* (%)						
Male	1 (17)	2 (50)	1 (33)	1 (33)	2 (67)	7 (37)
Female	5 (83)	2 (50)	2 (67)	2 (67)	1 (33)	12 (63)
Race/ethnicity, *N* (%)						
White	4 (67)	4 (100)	3 (100)	1 (33)	3 (100)	15 (79)
Hispanic	0	0	0	0	0	0
Black	1 (17)	0	0	1 (33)	0	2 (11)
Asian	1 (17)	0	0	1 (33)	0	2 (11)
ECOG performance status, *N* (%)						
0	1 (17)	0	1 (33)	2 (67)	1 (33)	5 (26)
1	5 (83)	4 (100)	2 (67)	1 (33)	2 (67)	14 (74)
Primary tumor, *N* (%)						
Ovarian	0	2 (50)	0	0	0	2 (11)
Breast	3 (50)	0	0	0	0	3 (16)
Colorectal cancer	0	0	1 (33)	1 (33)	1 (33)	3 (16)
Endometrial/fallopian	0	0	0	0	0	0
Lung	1 (17)	0	0	0	0	1 (5)
Neuroendocrine	0	0	1 (33)	1 (33)	0	2 (11)
Pancreas	0	0	1 (33)	0	1 (33)	2 (11)
Esophageal	0	0	0	0	0	0
Head and neck/salivary gland	1 (17)	0	0	1 (33)	0	2 (11)
Liver/cholangiocarcinoma	0	0	0	0	1 (33)	1 (5)
Sarcoma	0	1 (25)	0	0	0	1 (5)
Prostate	0	1 (25)	0	0	0	1 (5)
Others	1 (17)	0	0	0	0	1 (5)
Prior lines of systemic therapies, *N* (%)						
0–1	1 (17)	0	0	0	0	1 (5)
2–3	4 (67)	3 (75)	2 (67)	2 (67)	2 (67)	13 (68)
4–5	1 (17)	1 (25)	1 (33)	0	1 (33)	4 (21)
> 5	0	0	0	1 (33)	0	1 (5)
Median range	2.5 (1.0–4.0)	2.0 (2.0–4.0)	3.0 (3.0–5.0)	3.0 (2.0–6.0)	3.0 (2.0–4.0)	3.0 (1.0–6.0)

*N* number; *ECOG* Eastern Cooperative Oncology Group; *QW* once weekly; *BIW* twice weekly; *AUC* area under curve; *mg/m*^*2*^ milligrams per square meter; *D1 and 8* on days 1, and 8 of each cycle; *Q3W* every 3 week; *FOLFIRI* irinotecan with fluorouracil and folinic acid; *XELOX* capecitabine and oxaliplatin

^a^FOLFIRI—irinotecan of 180 mg/m^2^, 5-FU continuous infusion of 2400 mg/m^2^, 5-FU bolus of 400 mg/m^2^, and leucovorin of 400 mg/m^2^ on days 1, and 15

^b^XELOX—capecitabine was dosed at 900 mg/m^2^ orally (PO) divided into 2 doses on days 1–14, along with oxaliplatin of 130 mg/m^2^ IV Q3W

**Table 2:** Summary of treatment-related adverse events (TRAE) in all grades of severity

*N* (%)	Selinexor 60 mg QW/BIW + carboplatin 6 AUC Q3W (*N* = 6)		Selinexor 60 mg QW/BIW + doxorubicin 60 mg/m^2^ + cyclophosphamide 600 mg/m^2^ Q3W (*N* = 4)		Selinexor 40 mg QW + FOLFIRI^a^ (*N* = 3)		Selinexor 60 mg QW + irinotecan 125 mg/m^2^ D1 and 8 Q3W (*N* = 3)		Selinexor 40 mg QW + XELOX^b^ (*N* = 3)		All patients (*N* = 19)	
	All grades	Grade 3/4	All grades	Grade 3/4	All grades	Grade 3/4	All grades	Grade 3/4	All grades	Grade 3/4	All grades	Grade 3/4
Anemia	5 (83)	2 (33)	2 (50)	0	1 (33)	0	1 (33)	0	1 (33)	1 (33)	10 (53)	3 (16)
Leukopenia	4 (75)	1 (17)	3 (75)	3 (75)	3 (100)	0	1 (33)	0	2 (67)	1 (33)	13 (68)	5 (26)
Neutropenia	4 (75)	2 (33)	3 (75)	3 (75)	2 (67)	1 (33)	2 (67)	1 (33)	1 (33)	1 (33)	12 (63)	8 (42)
Thrombocytopenia	6 (100)	3 (50)	3 (75)	0	2 (67)	0	2 (67)	0	3 (100)	0	16 (84)	3 (16)
Constipation	0	0	0	0	0	0	0	0	0	0	0	0
Diarrhea	2 (33)	0	0	0	0	0	1 (33)	0	1 (33)	1 (33)	4 (21)	1 (5)
Nausea	4 (75)	0	4 (100)	0	1 (33)	0	1 (33)	0	3 (100)	0	13 (68)	0
Vomiting	4 (75)	0	3 (75)	0	0	0	1 (33)	0	2 (67)	0	10 (53)	0
Elevated AST/ALT	0	0	0	0	1 (33)	0	1 (33)	0	0	0	2 (11)	0
Mucositis	0	0	0	0	0	0	0	0	0	0	0	0
Fatigue	4 (75)	0	2 (50)	0	1 (33)	0	3 (100)	1 (33)	1 (33)	0	11 (58)	1 (5)
Anorexia	1 (17)	0	0	0	0	0	1 (33)	0	2 (67)	0	4 (21)	0
Hyponatremia	3 (50)	2 (33)	0	0	1 (33)	1 (33)	1 (33)	0	1 (33)	1 (33)	6 (32)	4 (21)
Hypomagnesemia	1 (17)	0	0	0	0	0	0	0	1 (33)	0	2 (11)	0
Hypoalbuminemia	0	0	0	0	0	0	0	0	0	0	0	0
Dyspnea	0	0	0	0	0	0	0	0	1 (33)	0	1 (5)	0
Cough	0	0	0	0	0	0	0	0	0	0	0	0
Elevated CPK	1 (17)	0	0	0	0	0	0	0	0	0	1 (5)	0
Infection or infestation	0	0	0	0	0	0	0	0	0	0	0	0
Elevated lipase	1 (17)	1 (17)	0	0	0	0	0	0	0	0	1 (5)	1 (5)

^a^FOLFIRI—irinotecan of 180 mg/m^2^, 5-FU continuous infusion of 2400 mg/m^2^, 5-FU bolus of 400 mg/m^2^, and leucovorin of 400 mg/m^2^ on days 1, and 15

^b^XELOX—capecitabine was dosed at 900 mg/m^2^ orally (PO) divided into 2 doses on days 1–14, along with oxaliplatin of 130 mg/m^2^ IV Q3W

*QW* once weekly; *BIW* twice weekly; *AUC* area under curve; *mg/m*^*2*^ milligrams per square meter; *D1 and 8* on days 1, and 8 of each cycle; *Q3W* every 3 weeks; *ALT* alanine aminotransferase; *AST* aspartate aminotransferase; *CPK* creatine phosphokinase; *FOLFIRI* irinotecan with fluorouracil and folinic acid; *5-FU* 5-fluorouracil; *XELOX* capecitabine and oxaliplatin

All patients enrolled had measurable disease, but 5 patients had not completed their first restaging scans due to earlier withdrawal of consent and toxicity. Fourteen patients were considered efficacy evaluable patients (Additional file 1: Figure S1). No patients had objective responses, however, 7 patients had SD. Of the 7 SD patients, 2 received selinexor with DC (ovarian cancer), 2 received selinexor with FOLFIRI [colorectal cancer (CRC); neuroendocrine carcinoma of the pancreas], 2 received selinexor with XELOX (CRC; cholangiocarcinoma), and 1 received selinexor with irinotecan (CRC). All had progressed on multiple prior lines of therapy including FOLFOX, FOLFIRI, cisplatin  +  gemcitabine, FOLFRINOX, temozolomide, everolimus, octreotide, capecitabine, sunitinib, and ziv-aflibercept. The median time-to-treatment failure (TTF) was 20 weeks (range, 6–29 weeks). Two patients who received selinexor with XELOX experienced disease control resulting in a DCR of 14%. A patient with CRC who progressed on prior 4 lines of therapies, had stable disease with TTF of 26 weeks. Another patient with cholangiocarcinoma who progressed on prior 3 lines of therapies including cisplatin  +  gemcitabine, FOLFRINOX, had stable disease with TTF of 29 weeks. The median PFS for all patients was 2.0 months (95% CI 1.2, 2.8) while the median OS for all patients was 5.2 months (95% CI 4.0, 11.2) (Additional file 1: Figure S2).

In conclusion, selinexor in combination with standard chemotherapy showed limited clinical activity with some toxicity. Although RP2D of selinexor was 40 mg QW in combination with XELOX or FOLFIRI, the study arms were not pursued for dose expansion due to toxicities and lack of response. Optimizing supportive care, proper use of growth factors, and aggressive measures on antiemetics strategies are important to mitigate TRAE.

## Supplementary Information

**Additional file 1:** **Table S1.** Summary of treatment-emergent adverse events in the phase I safety population. **Table S2.** Summary of treatment-emergent adverse events (TEAE) in all grades of severity. **Figure S1.** Waterfall plot of maximum change in tumor measurements (per RECIST v1.1) for evaluable patients. **Figure S2.** Kaplan-Meier plot showing progression-free survival (PFS) and overall survival (OS) for all treated patients.

## Data Availability

The datasets used and/or analyzed during the current study are available from the corresponding author on reasonable request.
