# Supplementary material for: Selinexor in combination with standard chemotherapy in patients with advanced or metastatic solid tumors
Source: Exp Hematol Oncol. 2021 Dec 29;10:59. doi: 10.1186/s40164-021-00251-0 (PMC8715578; doi:10.1186/s40164-021-00251-0)
Supplement: Supplementary file 1 — Additional file 1: Table S1. Summary of treatment-emergent adverse events in the phase I safety population. Table S2. Summary of treatment-emergent adverse events (TEAE) in all grades of severity. Figure S1. Waterfall plot of maximum change in tumor measurements (per RECIST v1.1) for evaluable patients. Figure S2. Kaplan-Meier plot showing progression-free survival (PFS) and overall survival (OS) for all treated patients. [file 40164_2021_251_MOESM1_ESM.docx]

**Table S1. Summary of treatment-emergent adverse events in the phase I safety population**

| **Measure, *n (%)*** | **Selinexor + carboplatin (*N=6*)** | **Selinexor + doxorubicin + cyclophosphamide (*N=4*)** | **Selinexor + FOLFIRI (*N=3*)** | **Selinexor + irinotecan (*N=3*)** | **Selinexor + XELOX**  **(*N=3*)** | **All Patients (*N=19*)** |
| --- | --- | --- | --- | --- | --- | --- |
| ≥ 1 TEAE | 6 (100) | 4 (100) | 3 (100) | 3 (100) | 3 (100) | 19 (100) |
| ≥ 1 TRAE | 6 (100) | 4 (100) | 3 (100) | 3 (100) | 3 (100) | 19 (100) |
| Grade 3/4 TEAE | 6 (100) | 4 (100) | 3 (100) | 2 (67) | 1 (33) | 16 (84) |
| Grade 3/4 TRAE | 6 (100) | 4 (100) | 3 (100) | 2 (67) | 1 (33) | 16 (84) |
| SAE | 0 | 1^a^ (25) | 2^c^ (67) | 0 | 1^e^ (33) | 4 (21) |
| ≥ 1 TRSAE | 0 | 1 (25) | 1 (33) | 0 | 1 (33) | 3 (16) |
| At least one DLT | 0 | 1^b^ (25) | 1^d^ (33) | 0 | 1^f^ (33) | 3 (16) |
| Discontinued due to |  |  |  |  |  |  |
| ≥ 1 TEAE | 1 (17) | 2 (50) | 0 | 1 (33) | 1 (33) | 5 (26) |

Abbreviations: N, number; TRAE, treatment-related adverse events; SAE, serious adverse events; TRSAE, treatment-related serious adverse events; DLT, dose limiting toxicity; FOLFIRI, 5-fluorouracil, leucovorin, and irinotecan; XELOX, capecitabine and oxaliplatin.

^a^ One patient had SAE from treatment-related grade 3 febrile neutropenia.

^b^ One patient receiving selinexor at 60 mg twice weekly experienced DLTs with grade 3 leukopenia and grade 4 neutropenia.

^c^ Two patients had SAE; one patient had treatment-related grade 3 febrile neutropenia while the other had grade 2 pancreatitis unrelated to treatment.

^d^ One patient receiving selinexor at 40 mg once weekly experienced a DLT with grade 3 febrile neutropenia.

^e^ One patient had SAE from treatment-related grade 3 diarrhea and grade 3 dyspnea and skin infection unrelated to treatment.

^f^ One patient receiving selinexor at 40 mg once weekly experienced a DLT with grade 3 diarrhea.

**Table** **S2. Summary of treatment-emergent adverse events (TEAE) in all grades of severity**

|  | **Selinexor 60mg QW/ BIW + carboplatin 6 AUC Q3W**  **(*N=6*)** | | **Selinexor 60mg QW/ BIW + doxorubicin 60 mg/m^2^ + cyclophosphamide 600mg/m^2^ Q3W**  **(*N=4*)** | | **Selinexor 40mg QW + FOLFIRI***  **(*N=3*)** | | **Selinexor 60mg QW+ irinotecan 125 mg/m^2^ D1 & 8 Q3W**  **(*N=3*)** | | **Selinexor 40mg QW + XELOX****  **(*N=3*)** | | **All Patients**  **(*N=19*)** | | |
| --- | --- | --- | --- | --- | --- | --- | --- | --- | --- | --- | --- | --- | --- |
|  | All Grades | Grade 3/4 | All Grades | Grade 3/4 | All Grades | Grade 3/4 | All Grades | Grade 3/4 | All Grades | Grade 3/4 | All Grades | Grade 3/4 |  |
| *n (%)* |  |  |  |  |  |  |  |  |  |  |  |  |  |
| Anemia | 5 (83) | 2 (33) | 2 (50) | 0 | 1 (33) | 0 | 2 (67) | 1 (33) | 1 (33) | 1 (33) | 11 (58) | 4 (21) |  |
| Leukopenia | 4 (75) | 1 (17) | 3 (75) | 3 (75) | 3 (100) | 0 | 1 (33) | 0 | 2 (67) | 1 (33) | 13 (68) | 5 (26) |  |
| Neutropenia | 4 (75) | 2 (33) | 3 (75) | 3 (75) | 2 (67) | 1 (33) | 2 (67) | 1 (33) | 1 (33) | 1 (33) | 12 (63) | 8 (42) |  |
| Thrombocytopenia | 6 (100) | 3 (50) | 3 (75) | 0 | 2 (67) | 0 | 2 (67) | 0 | 3 (100) | 0 | 16 (84) | 3 (16) |  |
| Constipation | 0 | 0 | 0 | 0 | 1 (33) | 0 | 1 (33) | 0 | 1 (33) | 0 | 3 (16) | 0 |  |
| Diarrhea | 2 (33) | 0 | 0 | 0 | 1 (33) | 0 | 3 (100) | 0 | 1 (33) | 1 (33) | 7 (37) | 1 (5) |  |
| Nausea | 4 (75) | 0 | 4 (100) | 0 | 1 (33) | 0 | 1 (33) | 0 | 3 (100) | 0 | 13 (68) | 0 |  |
| Vomiting | 4 (75) | 0 | 3 (75) | 0 | 0 | 0 | 1 (33) | 0 | 2 (67) | 0 | 10 (53) | 0 |  |
| Elevated AST/ALT | 0 | 0 | 0 | 0 | 2 (67) | 0 | 3 (100) | 0 | 1 (33) | 0 | 6 (32) | 0 |  |
| Mucositis | 0 | 0 | 0 | 0 | 0 | 0 | 0 | 0 | 1 (33) | 0 | 1 (5) | 0 |  |
| Fatigue | 4 (75) | 0 | 2 (50) | 0 | 1 (33) | 0 | 3 (100) | 1 (33) | 1 (33) | 0 | 11 (58) | 1 (5) |  |
| Anorexia | 1 (17) | 0 | 0 | 0 | 0 | 0 | 2 (67) | 0 | 2 (67) | 0 | 5 (26) | 0 |  |
| Hyponatremia | 3 (50) | 2 (33) | 0 | 0 | 1 (33) | 1 (33) | 1 (33) | 0 | 1 (33) | 1 (33) | 6 (32) | 4 (21) |  |
| Hypomagnesemia | 1 (17) | 0 | 0 | 0 | 1 (33) | 0 | 2 (67) | 0 | 1 (33) | 0 | 5 (26) | 0 |  |
| Hypoalbuminemia | 0 | 0 | 0 | 0 | 1 (33) | 0 | 0 | 0 | 1 (33) | 0 | 2 (11) | 0 |  |
| Dyspnea | 0 | 0 | 0 | 0 | 0 | 0 | 0 | 0 | 2 (67) | 1 (33) | 2 (11) | 1 (5) |  |
| Cough | 0 | 0 | 0 | 0 | 0 | 0 | 0 | 0 | 2 (67) | 0 | 2 (11) | 0 |  |
| Elevated CPK | 1 (17) | 0 | 0 | 0 | 1 (33) | 0 | 0 | 0 | 1 (33) | 0 | 3 (16) | 0 |  |
| Infection or infestation | 0 | 0 | 0 | 0 | 1 (33) | 0 | 1 (33) | 1 (33) | 2 (67) | 1 (33) | 4 (21) | 2 (11) |  |
| Elevated Lipase | 1 (17) | 1 (17) | 0 | 0 | 2 (67) | 1 (33) | 0 | 0 | 0 | 0 | 3 (16) | 2 (11) |  |

* FOLFIRI - irinotecan of 180 mg/m^2^, 5-FU continuous infusion of 2400 mg/m^2^, 5-FU bolus of 400 mg/m^2^, and leucovorin of 400 mg/m^2^ on days 1, and 15

** XELOX - capecitabine was dosed at 900 mg/m^2^ orally (PO) divided into 2 doses on days 1-14, along with oxaliplatin of 130 mg/m^2^ IV Q3W.

Abbreviations: QW, once weekly; BIW, twice weekly; AUC, area under curve; mg/m^2^, milligrams per square meter; D1 & 8, on days 1, and 8 of each cycle; Q3W, every 3 week; ALT, alanine aminotransferase; AST, aspartate aminotransferase; CPK, creatine phosphokinase; FOLFIRI, 5-fluorouracil, leucovorin, and irinotecan; XELOX, capecitabine and oxaliplatin.

**Figure S1. Waterfall plot of maximum change in tumor measurements (per RECIST v1.1) for evaluable patients**


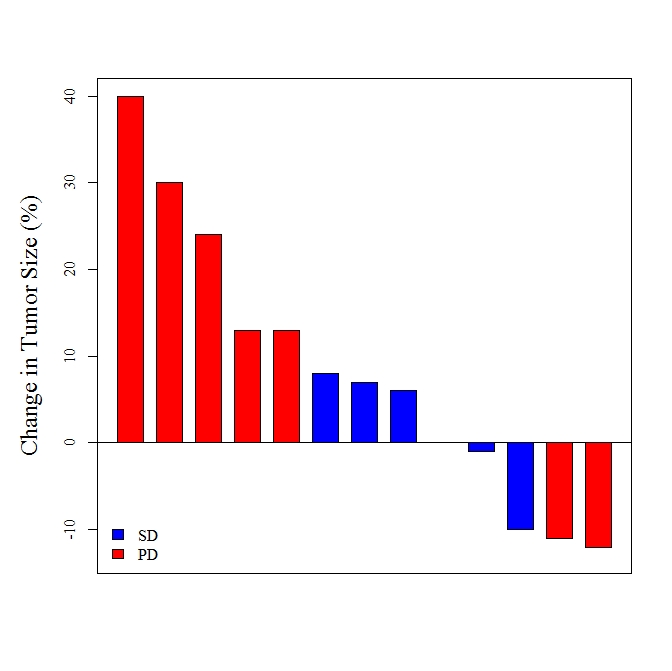


Abbreviation: RECIST v1.1, response evaluation criteria in solid tumors version 1.1; SD, stable disease; PD, progressive disease.

^a^One patient with SD in the waterfall plot had a change of zero and one patient with SD had missing values for tumor change.

**Figure S2. Kaplan-Meier plot showing progression-free survival (PFS) and overall survival (OS) for all treated patients**

Abbreviations: n, number; S, selinexor; C, carboplatin; I, irinotecan; DC, cyclophosphamide and doxorubicin; F (FOLFIRI), 5-fluorouracil, leucovorin, and irinotecan; X (XELOX), capecitabine and oxaliplatin.

(Trial registration: NCT02419495. Registered 14 April 2015, https://clinicaltrials.gov/ct2/show/NCT02419495)

**TRIAL INFORMATION**

• ClinicalTrials.gov Identifier: NCT02419495

• Sponsor(s): Karyopharm Therapeutics

**Funding**

- Karyopharm Therapeutics, Clinical and Translational Sciences Award (1UL1TR003167) (NIH/NCATS), and MD Anderson Cancer Support Grant (P30CA016672) (NIH-NCI)

**Patients**

Adult (age ≥18 years) patients with histologically documented, advanced or metastatic solid tumors (excluding brain tumors) whose tumors were unresponsive or had relapsed following prior systemic therapy or where the addition of selinexor to standard chemotherapy deemed appropriate and acceptable were eligible. Other key inclusion criteria included Eastern Cooperative Oncology Group (ECOG) performance status of 0 or 1 and adequate organ function. The number of prior treatments was not limited. Patients in the study had to have at least one measurable target lesion as defined by Response Evaluation Criteria in Solid Tumors (RECIST v1.1) [1, 2] criteria for solid tumors, except for patients with castrate resistant prostate cancer (CRPC) where prostate cancer working group 2 (PCWG2) criteria was utilized.[3] Key exclusions were patients with primary CNS tumor or active CNS tumor involvement, evidence of complete or partial bowel obstruction or needing total parenteral nutrition, prior treatment with an agent targeting the exportin, and unstable cardiovascular functions.

The primary objective was to establish the safety and tolerability of selinexor when given in combination with standard chemotherapy regimens; secondary objectives included determining the disease control rate (DCR) and progression free survival (PFS) of selinexor administered with standard chemotherapy treatments. The primary efficacy parameter was the tolerability according to National Cancer Institute Common Terminology Criteria for Adverse Events (CTCAE) version 4.03 and the secondary parameters were clinical benefit rate (CBR; percentage of complete response [CR], partial response [PR] plus stable disease [SD]), DCR (percentage of CR, PR plus SD for at least 6 months, assessed according to RECIST 1.1 criteria), the objective tumor response rate (CR+PR), assessed according to RECIST 1.1 criteria and PFS defined as the time between the cycle 1 start date and the date of disease progression or death, whichever is reported first.

**Study design and treatment**

This open-label, single-center, multi-arm phase 1b study of selinexor in combination with standard chemotherapy was conducted to determine the dose-limiting toxicities (DLTs) and maximum tolerated dose (MTD) of selinexor and to further explore the safety and tolerability of the MTD in patients with advanced or metastatic solid tumors (ClinicalTrial.gov identifier: NCT02419495). The study was conducted in multiple arms by using a standard 3 + 3 design and a “basket-type” expansion. The study protocol was approved by the Institutional Review Board or Independent Ethics Committee at MD Anderson Cancer Center and was conducted in accordance with the Declaration of Helsinki, Good Clinical Practice, and all local and federal regulatory guidelines. All patients signed informed consent prior to enrolling onto the study.

Selinexor with carboplatin, irinotecan, doxorubicin and cyclophosphamide (DC), irinotecan with fluorouracil and folinic acid (FOLFIRI), and capecitabine and oxaliplatin (XELOX), were employed as separate parallel arms. Standard single agent chemotherapy was dosed as followings; carboplatin was dosed at AUC6 intravenously (IV) every 3 weeks (Q3W), and irinotecan was used at 125 mg/m^2^ on days 1 and 8 of 21-day cycle. Standard combination chemotherapy regimens were dosed as followings; doxorubicin was dosed at 60 mg/m^2^ IV Q3W with cyclophosphamide 600 mg/m^2^ IV Q3W, FOLFIRI was used on days 1, and 15 (irinotecan of 180 mg/m^2^, 5-FU continuous infusion of 2400 mg/m^2^, 5-FU bolus of 400 mg/m^2^, and leucovorin of 400 mg/m^2^), and capecitabine was dosed at 900 mg/m^2^ orally (PO) divided into 2 doses on days 1-14, along with oxaliplatin of 130 mg/m^2^ IV Q3W. Selinexor was dosed at 60 mg twice weekly (BID) PO on days 1, 3, 8, and 10 of each 21-day cycle as well as 40-60 mg once weekly (QW) on days 1, 8, and 15. Selinexor was dosed at either 40 mg once weekly on days 1, 8, and 15 in combination with XELOX, and FOLFIRI, or 60 mg once weekly in combination with irinotecan, or DC. Selinexor was dosed at 60 mg twice weekly on days 1, 3, 8, and 10 of each 21-day cycle or once weekly on days 1, 8, and 15 in combination with carboplatin.

**Study assessments**

Tumor response was assessed using RECIST v1.1. Baseline imaging was done within 30 days of treatment initiation. Repeat imaging (using the same methodology as at baseline) was obtained every 9 weeks. Treatment-emergent adverse events (TEAEs) and treatment-related adverse events (TRAEs) were graded using the Common Terminology Criteria for Adverse Events (CTCAE) version 4.03. DLT was defined as any selinexor-related grade 4 hematologic adverse event, grade ≥3 thrombocytopenia associated with clinically significant bleeding, febrile neutropenia or non-hematologic adverse event grade ≥ 3 in severity per CTCAE (v 4.03) despite optimal supportive medications, excluding electrolyte abnormalities that are reversible, asymptomatic or hair loss which is not dose-limiting. The MTD was defined as the highest dose level at which ≤ 33% of patients experience DLTs during cycle 1. After the MTD was defined in each schedule, the study was extended to include additional evaluable patients at the MTD. A safety monitoring committee comprised of investigators and the study sponsor reviewed all safety information and made consensus decisions about dose escalation.

**Statistical methods**

Patient characteristics, TEAEs, TRAEs, tumor response, and time-to-treatment failure (TTF) were summarized using descriptive statistics. PFS time was computed from cycle 1 start date to the date of disease progression or death (if the patient died without disease progression), or the last evaluation date. Patients who were alive and did not experience progression of disease at the last follow-up date were censored. Overall survival time (OS) was computed from cycle 1 start date to the last-known vital sign. Patients alive at the last follow-up date were censored. The Kaplan-Meier method was used to estimate PFS and OS. All statistical analyses were performed using SAS 9.4 for Windows (Copyright © 2002-2012 by SAS Institute Inc., Cary, NC).

**References**

1 Eisenhauer EA, Therasse P, Bogaerts J, et al. New response evaluation criteria in solid tumours: Revised recist guideline (version 1.1). Eur J Cancer 2009;45:228-247.

2 Nishino M, Jagannathan JP, Ramaiya NH, et al. Revised recist guideline version 1.1: What oncologists want to know and what radiologists need to know. AJR American journal of roentgenology 2010;195:281-289.

3 Sonpavde G, Pond GR, Armstrong AJ, et al. Radiographic progression by prostate cancer working group (pcwg)-2 criteria as an intermediate endpoint for drug development in metastatic castration-resistant prostate cancer. BJU Int 2014;114:E25-e31.
